# Sirtuin 2 Alleviates Chronic Neuropathic Pain by Suppressing Ferroptosis in Rats

**DOI:** 10.3389/fphar.2022.827016

**Published:** 2022-03-23

**Authors:** Xiaojiao Zhang, Tao Song, Mengnan Zhao, Xueshu Tao, Bohan Zhang, Cong Sun, Pinying Wang, Kunpeng Wang, Lin Zhao

**Affiliations:** Department of Pain Medicine, The First Hospital of China Medical University, Shenyang, China

**Keywords:** chronic neuropathic pain, SIRT2, ferroptosis, iron accumulation, lipid peroxidation

## Abstract

Neuropathic pain (NP) is chronic and associated with poor effects of general analgesia. It affects patients’ health and quality of life. The apoptotic process of lipid peroxidation caused by iron overload is called ferroptosis, which may be associated with nervous system disease. A recent study has found that sirtuin 2 (SIRT2) achieves a neuroprotective effect by suppressing ferroptosis. Herein, we aimed to examine whether SIRT2 regulated spared nerve injury (SNI)-induced NP by suppressing ferroptosis in rats. A rat model of NP was induced in adult male Sprague-Dawley rats weighing 200–250 g. Mechanical allodynia was observed from the first day after SNI and continued for 14 days. Compared with age-matched control rats, the expression of SIRT2 and ferroportin 1 (FPN1) decreased in the L4-6 spinal cord of the SNI-induced NP rats. In addition, we observed that the levels of both iron and anti-acyl-coenzyme A synthetase long-chain family member 4 (ACSL4) were significantly increased in the spinal cord after SNI, while the expression of glutathione peroxidase 4 (GPX4) was decreased. Furthermore, an intrathecal injection of SIRT2 overexpressed recombinant adenovirus, which upregulated the expression of SIRT2, attenuated mechanical allodynia, enhanced the level of FPN1, inhibited intracellular iron accumulation, and reduced oxidant stress levels, thereby reversing the changes to ACSL4 and GPX4 expression in the SNI rats. This evidence suggests that SIRT2-targeted therapeutics may help relieve the symptoms of chronic NP.

## Introduction

Neuropathic pain (NP) refers to the pain caused by neuropathy and damage related to hyperalgesia and paresthesia observed in any part of the peripheral or central nervous systems. Trigeminal neuralgia, postherpetic neuralgia, and diabetic peripheral neuropathy are common chronic pain types that are presented in the clinic. NP is a chronic condition affecting patients’ general health and quality of life; in addition, it is associated with a high economic burden (Carrasco et al., 2018). Despite recent research advances, the pathophysiological mechanism of NP remains unclear, and available treatments are still not satisfactory.

Several preclinical and clinical studies have shown that NP mechanisms are related to ferroptosis (Guo et al., 2021; Jia et al., 2021; Wang et al., 2021) Ferroptosis is different from cell apoptosis in that it highly depends on iron. The cytomembrane is not damaged during apoptosis; however, with the increase in the levels of reactive oxygen species (ROS) during ferroptosis, the integrity of the cell membrane is destroyed (Qiu et al., 2020). There are two types of antioxidant systems that eliminate ROS in the body; these systems are divided into enzyme- and non-enzyme-related types; the enzyme-related types include superoxide dismutase (SOD), catalase, and glutathione peroxidase (GPx) systems; the non-enzyme-related types mainly include the reduction of glutathione (GSH) and vitamin C/E systems. High levels of GSH, GPx, and SOD can downregulate ROS production.

In this context, the deficiency of GSH and functional loss of glutathione peroxidase 4 (GPX4, the only member of GPx family that has an antagonistic effect on membrane lipid peroxidation) causes ferroptosis (Brigelius-Flohé and Maiorino, 2013; Yang et al., 2014). Anti-acyl-coenzyme A synthetase long-chain family member 4 (ACSL4) is considered to play a crucial role in ferroptosis (Ding et al., 2021). In the absence of ACSL4, lipid peroxidation substrate levels decrease *in vivo*, which prevents ferroptosis (Yuan et al., 2016a; Doll et al., 2017). Ferroportin 1 (FPN1) is a type of integral membrane protein that exports iron from cells to plasma (Abboud and Haile, 2000; Donovan et al., 2000; McKie et al., 2000). The importance of FPN1 in cellular iron homeostasis and some other diseases such as cancer has attracted more and more attention. Consequently, if the levels of FPN1 are reduced, iron cannot be transported out of the cell, leading to intracellular iron accumulation, which facilitates ferroptosis.

Sirtuin is a highly conserved deacetylase. The sirtuin family has seven members, SIRT1–SIRT7 (Kong et al., 2017). They are involved in regulating the oxidative stress response in many diseases (Singh et al., 2018). Sirtuin 2 (SIRT2, the only member of sirtuin family that is found in the cytoplasm) is involved in regulating neurological diseases. Previous studies have found that SIRT2 has a neuroprotective effect in rats with chronic constriction injury (CCI) (Zhang and Chi, 2018). Moreover, SIRT2 regulates chronic NP through the nuclear factor erythroid 2-related factor 2 (NRF2) in rats (Zhao et al., 2021). A recent study has shown that inhibiting ferroptosis attenuated NP in rats (Guo et al., 2021). Meanwhile, another recent study has demonstrated that SIRT2 alleviated nerve injury by inhibiting p53-mediated ferroptosis in a mouse model of traumatic brain injury (TBI) (Gao et al., 2021). Overall, this evidence suggests that SIRT2 may alleviate NP by inhibiting ferroptosis; consequently, understanding the physiological mechanism of SIRT2 may help inform clinical treatment of NP. Herein, we aimed to examine whether SIRT2 regulated spared nerve injury (SNI)-induced NP by suppressing ferroptosis in rats.

## Materials and Methods

### Animals

About 70 adult male Sprague-Dawley rats weighing 200–250 g were used in the animal model. These rats were purchased from Beijing Charles River Experimental Animal Technology Co., Ltd. The rats were raised in separate cages at approximately 22°C and 50–60% humidity with free access to enough water and food. The animal experiment protocol was reviewed and approved by the Institutional Animal Care and Use Committee (IACUC) of China Medical University.

### Establishment of the Neuropathic Pain Model

The rat model of NP was induced by conducting unilateral SNI, as previously described (Decosterd and Woolf, 2000; Guo et al., 2019). In short, rats were anesthetized with 5% isoflurane by a small animal anesthesia machine and maintained with 2–3% isoflurane. The rats were placed in a left decubitus position; approximately 1 cm below the right iliac bone, the three branches of the sciatic nerve were exposed: the tibial nerve, common peroneal nerve, and sural nerve. The thicker nerves, common peroneal nerve, and tibial nerve were ligated and removed. During this process, care was taken to preserve the integrity of the sural nerve. The muscle and skin layers were sutured with 3.0 silk, and the surgical incision was disinfected. In the sham operation, the sciatic nerve and its three branches were exposed but not ligated. Rats subjected to the sham operation were used as the control groups.

### Administration of Recombinant Adenoviruses

Recombinant adenoviruses overexpressing SIRT2 (Ad-SIRT2) were purchased from Shanghai GeneChem Co., Ltd. In brief, rats were anesthetized with 5% isoflurane inhalation and maintained with 2–3% isoflurane. The rats were placed prone on the operating table, about 20 µl of 1 × 10^8^ pfu of Ad-SIRT2 or Ad-control was injected vertically into the interspace of L5-6 the spinous process with a microinjection syringe. Correct intrathecal injection was identified by the tail-flicking movement when the needle was inserted into the subarachnoid space. For biological testing, the L4-6 lumbar enlargement was removed and tested.

### Experiment Protocols

#### Protocol I

To detect the changes in pain behavior and the expression of SIRT2 and FPN1 in rat spinal cord after SNI, the rats were divided into the sham group (*n* = 5) and SNI group (*n* = 30). SNI was performed in the SNI group, whereas the sham group had their nerves exposed but did not undergo further intervention. Behavioral tests were performed on days 0, 1, 3, 7, 10, and 14 post-SNI. Five rats in the SNI group were killed after PWT was measured at each time point, and rats in the sham group were killed at the last time point. Protein was removed from the L4-6 lumbar enlargement of the spinal cord for biological tests on day 14 post-SNI. In addition, rats in the sham group and SNI group (*n* = 5 each) were used for immunofluorescence study to detect the localization and changes in SIRT2 and FPN1 in the spinal cord.

#### Protocol II

In order to further explore the role of SIRT in regulating neuropathic pain and ferroptosis in rats, the rats were divided into four groups (*n* = 5 each). SNI was performed in the SNI group, whereas the sham group had their nerves exposed but did not undergo further intervention. One day before the SNI, Ad-SIRT2 or Ad-control was injected intrathecally with a microinjection syringe in the Ad-SIRT2 group or Ad-control group respectively. Behavioral tests were performed on days 0, 1, 3, and 7 post-SNI. Tissue samples were harvested on day 7 post-SNI.

### Behavioral Assessment

**FIGURE 1:**
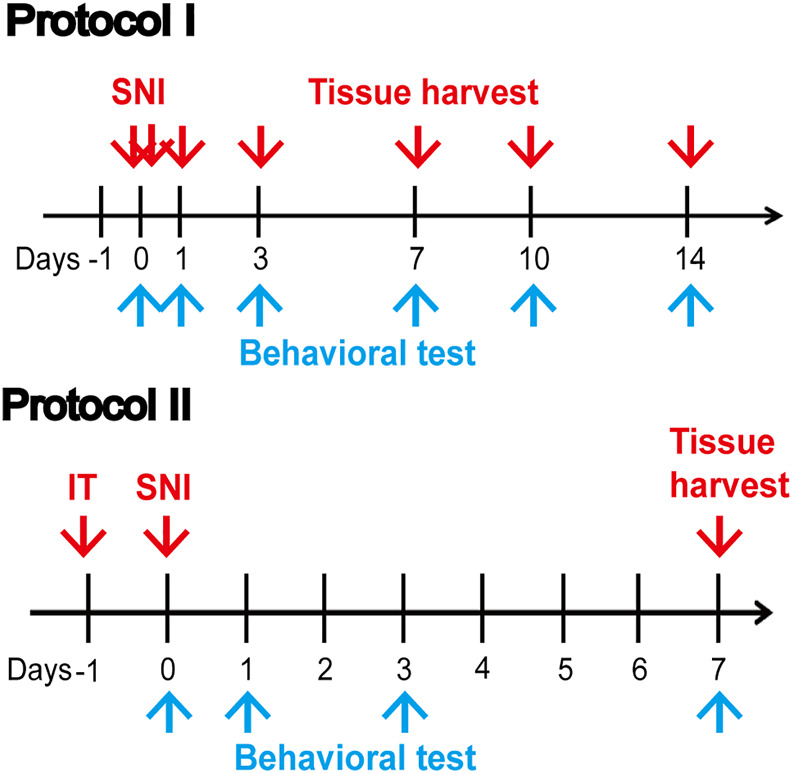
Illustrative diagram of the experiment. Protocol I: Behavioral tests were performed on days 0, 1, 3, 7, 10, and 14 post-SNI. Protein was removed from the L4-6 lumbar enlargement of the spinal cord for biological tests on day 14 post-SNI. Protocol II: A day before SNI, Ad-SIRT2 or Ad-control was injected intrathecally. Behavioral tests were performed on days 0, 1, 3, and 7 post-SNI. Tissue samples were harvested on day 7 post-SNI. SNI: spared nerve injury; IT: intrathecal injection of SIRT2 adenovirus (Ad-SIRT2) or Ad-control; blue arrow: Behavioral test.

Mechanical allodynia was measured by detecting the paw withdrawal threshold (PWT) stimulated by von Frey filaments, as previously described (Chaplan et al., 1994; Guo et al., 2019). Pain thresholds were measured blindly (Figure 1). Rats were housed separately in a cage with a wire mesh floor for at least 15 min before mechanical allodynia was measured. We stimulated the mid-plantar skin of the right hind paw of the rats with a medium-strength von Frey fiber filament (0.16, 0.2, 0.4, 1.0, 1.4, 2.0, 4.0, 6.0, 8.0, 10.0, 15.0 g). Acute retraction of the rat hind paw is considered to be a positive reaction. If the rat does not have this positive reaction, the filament was increased by one level; if a positive reaction occurs, a smaller level of filament was applied. The lowest level required to cause a positive reaction was recorded as PWT (g). The interval between detections was at least 30 s to eliminate the effects of the previous stimulus.

### Iron Content Assay

Tissue iron assay kits (A039-2-1, Jiancheng Bioengineering Institute, Nanjing, China) were applied to measure the iron content in the spinal cord according to the manufacturer’s instructions. The tissues were weighed accurately, and pre-cooled physiological saline was added at a weight (g) to volume (ml) ratio of 1:10. The centrifuge was used at 2,500 rpm for 10 min. The samples were collected according to the manufacturer’s instructions, and the absorbent optical density (OD) value of each sample was examined at a wavelength of 520 nm.

### Lipid Peroxidation Assays

Lipid peroxidation kits were used to measure the levels of malondialdehyde (MDA, A003-1, Jiancheng Bioengineering Institute, Nanjing, China) and SOD (A001-1, Jiancheng Bioengineering Institute, Nanjing, China). The tissues were weighed accurately, treated with a high-speed grinder, and centrifuged for 10 min at 2,500 rpm, and the supernatant was then taken for examination according to the manufacturer’s instructions. We detected the absorbent OD value of MDA at a wavelength of 532 nm and that of SOD at a wavelength of 550 nm.

### Western Blot Analysis

Levels of SIRT2, FPN1, GPX4 and ACSL4 proteins were detected by western blotting. Protein lysates were prepared from the spinal cord (L4-6). Subsequently, 48 μg protein samples were separated using a 12% gradient sodium dodecyl sulfate polyacrylamide electrophoresis (SDS-PAGE) gel and transferred to polyvinylidene difluoride (pore size of 0.45 μm) membrane (Bio-Rad). The membranes were blocked in non-fatty dry milk for 2 h at 37°C and then incubated with primary antibodies, including anti-SIRT2 (1:500, ab51023, Abcam), anti-FPN1 (1:1,000, NBP1-21502, Novus), anti-NRF2 (1:1,000, ab137550, Abcam), anti-GPX4 (1:5,000, ab125066, Abcam), anti-ACSL4 (1:10,000, ab155282, Abcam), and anti-GAPDH (1:10,000, 60004-1-Ig, Proteintech) antibodies at 4°C overnight, and incubated with horseradish peroxidase-conjugated secondary antibodies for 1 h at room temperature. We analyzed the density of protein bands using the NIHA ImageJ software.

### Immunofluorescence Analysis

Rats were perfused transcardially with 0.1 M phosphate-buffered saline (PBS), and the spinal cord (L4-6) tissue was fixed in 4% paraformaldehyde. After 24 h, the fluid was changed to 20% sucrose in 0.1 M PBS, and then the fluid was changed to 30% sucrose in 0.1 M PBS for 24 h to achieve extensive dehydration. The spinal cord specimens with a thickness of 14 μm were prepared by a freezing microtome (Leica, Germany). After blocking with 5% non-fatty dry milk for 1 h at room temperature, the sections were incubated with primary to rabbit anti-SIRT2 (1: 400, ab51023, Abcam) or rabbit anti-FPN1 (1:500, NBP1-21502, Novus), and either mouse anti-NeuN antibody (a neuronal marker, 1:400, ab104224, Abcam) or mouse anti-CD11b (1:400,CD11b, 1:400, MCA275R, BIO-RAD) overnight at 4°C, followed by the administration of Alexa Fluor 488 goat anti-mouse secondary antibody (1:2,000, Thermo Fisher Scientific, United States), and goat anti-rabbit IgG (H+L) CY3-conjugated antibody (1:400, S0011, Affinity Bioscience, Jiangsu, China). The double-stained sections were captured by a confocal laser-scanning microscope (Leica, Germany).

### Statistical Analysis

All data are expressed as the mean ± standard error (mean ± SE), and GraphPad Prism 8 software was used for statistical analysis. Differences between groups were analyzed by one-way or two-way analysis of variance (ANOVA), followed by Tukey’s multiple comparison tests. Differences were considered statistically significant at *p*-values of <0.05.

## Results

### The PWT was Significantly Decreased and SIRT2 and FPN1 Levels were Downregulated in SNI Rats

**FIGURE 2:**
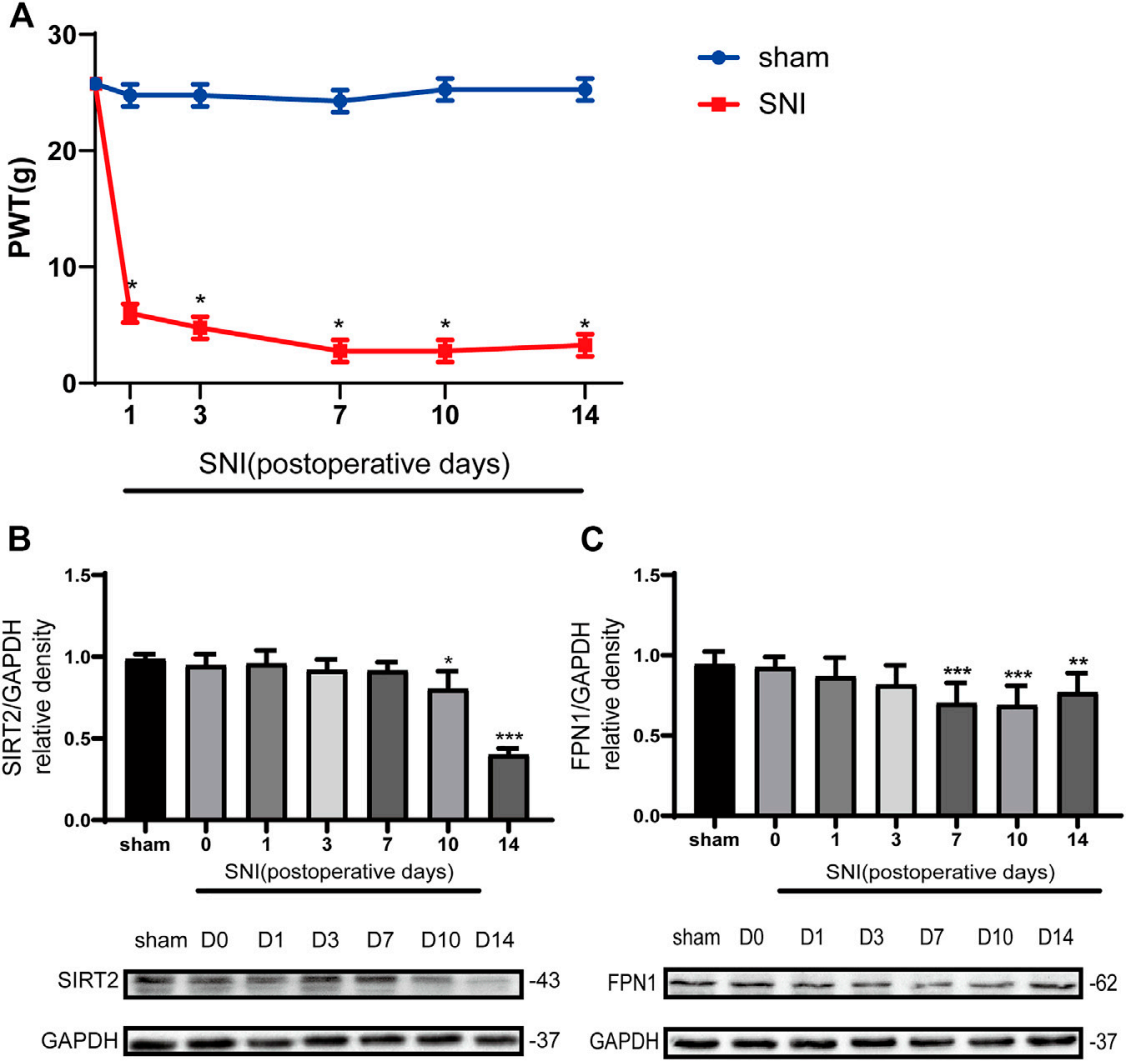
The paw withdrawal threshold (PWT) decreased in the SNI rats, and the levels of SIRT2 and FPN1 were downregulated in the SNI rats. **(A)** The PWT values to mechanical stimulation on days 0, 1, 3, 7, 10, and 14 after sham operation or SNI. **(B)** Time-dependent changes in the expression of SIRT2 in the spinal cord of the SNI rats. **(C)** Time-dependent changes in the expression of FPN1 in the spinal cord of the SNI rats. The rats in the sham operation group were used as controls. Values are expressed as the mean ± SE (*n* = 5 per group). *p-values of <0.05, **p-values of <0.01, and ***p-values of <0.001 for comparisons with values of the sham rats or SNI rats at baseline (day 0). SNI, spared nerve injury; SIRT2, sirtuin 2; FPN1, ferroportin 1.

The rates of mechanical allodynia were similar across the groups at baseline (Figure 2A). The PWT values of the SNI group began to decrease from day 1 post-SNI; this decrease in values lasted for up to 2 weeks, relative to the values observed in the sham control group and SNI group at baseline. The lowest PWT value was measured on day 7 in the SNI group.

The Western blot analysis showed that the expression of SIRT2 in the SNI group significantly decreased on days 10 and 14 compared with that in the sham group (Figure 2B). In addition, the expression of FPN1 in the SNI group decreased gradually from day 1 to day 14 compared with that in the sham group. A significant reduction in FPN1 expression in the SNI group was observed on day 7, 10, and 14 compared with the values observed in the sham group. This decrease was most significant on day 7 (Figure 2C).

**FIGURE 3:**
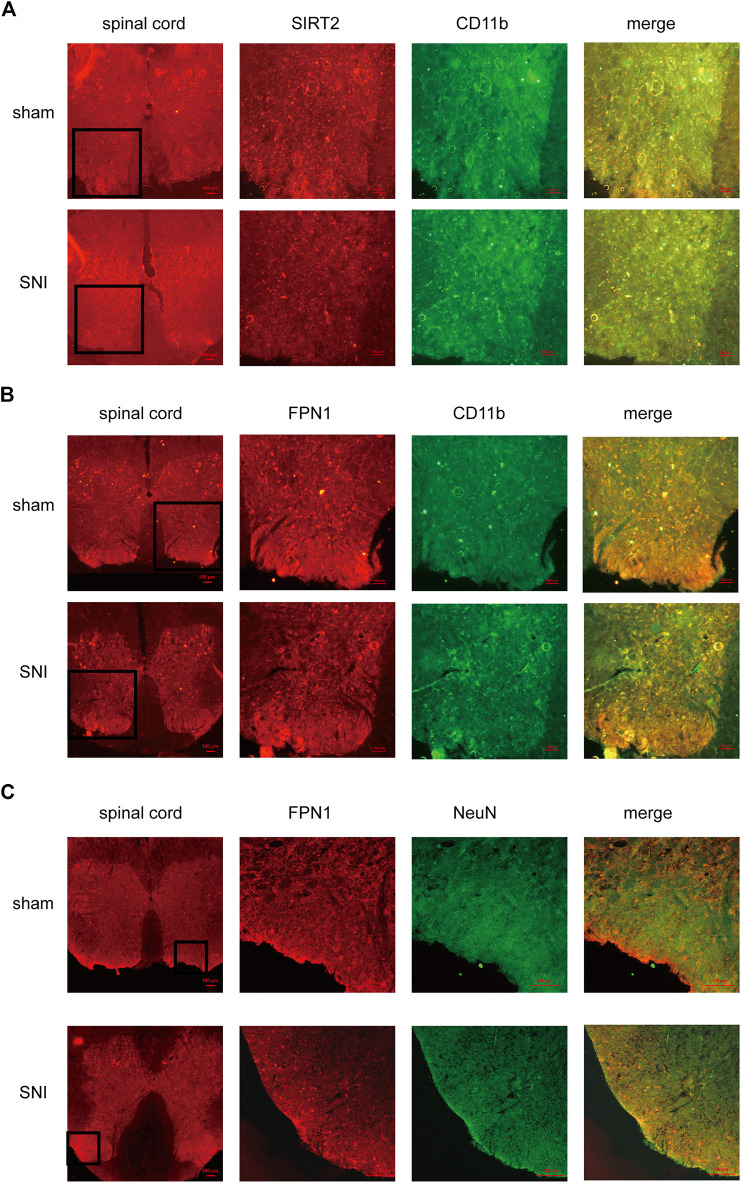
The cellular localization of SIRT2 and FPN1 in the spinal cord. **(A)** SIRT2 levels decreased in the microglia of SNI rats. **(B**,**C)** FPN1 levels decreased in the microglia and neurons of SNI rats. The image in the first column on the left shows the overall outline of the spinal cord. Representative confocal images show the results of double immunofluorescence staining of SIRT2 or FPN1 (red) in the spinal dorsal horn; CD11b, a microglia marker (green) or NeuN, a neuronal marker (green) in the sham group and SNI group. Scale bars, 100 μm. SNI, spared nerve injury; SIRT2, sirtuin 2; FPN1, ferroportin 1.

Immunofluorescence was applied to detect the cellular localization of SIRT2 and FPN1. The expression of SIRT2 was significantly decreased in the microglia in the spinal dorsal horn of the SNI rats (Figure 3A). In addition, the expression of FPN1 decreased in the microglia and neurons of the spinal cord horn in the SNI rats (Figures 3B,C).

### SIRT2 Adenovirus Attenuates Mechanical Allodynia, Upregulates FPN1 Expression, and Reduces Iron Accumulation

**FIGURE 4:**
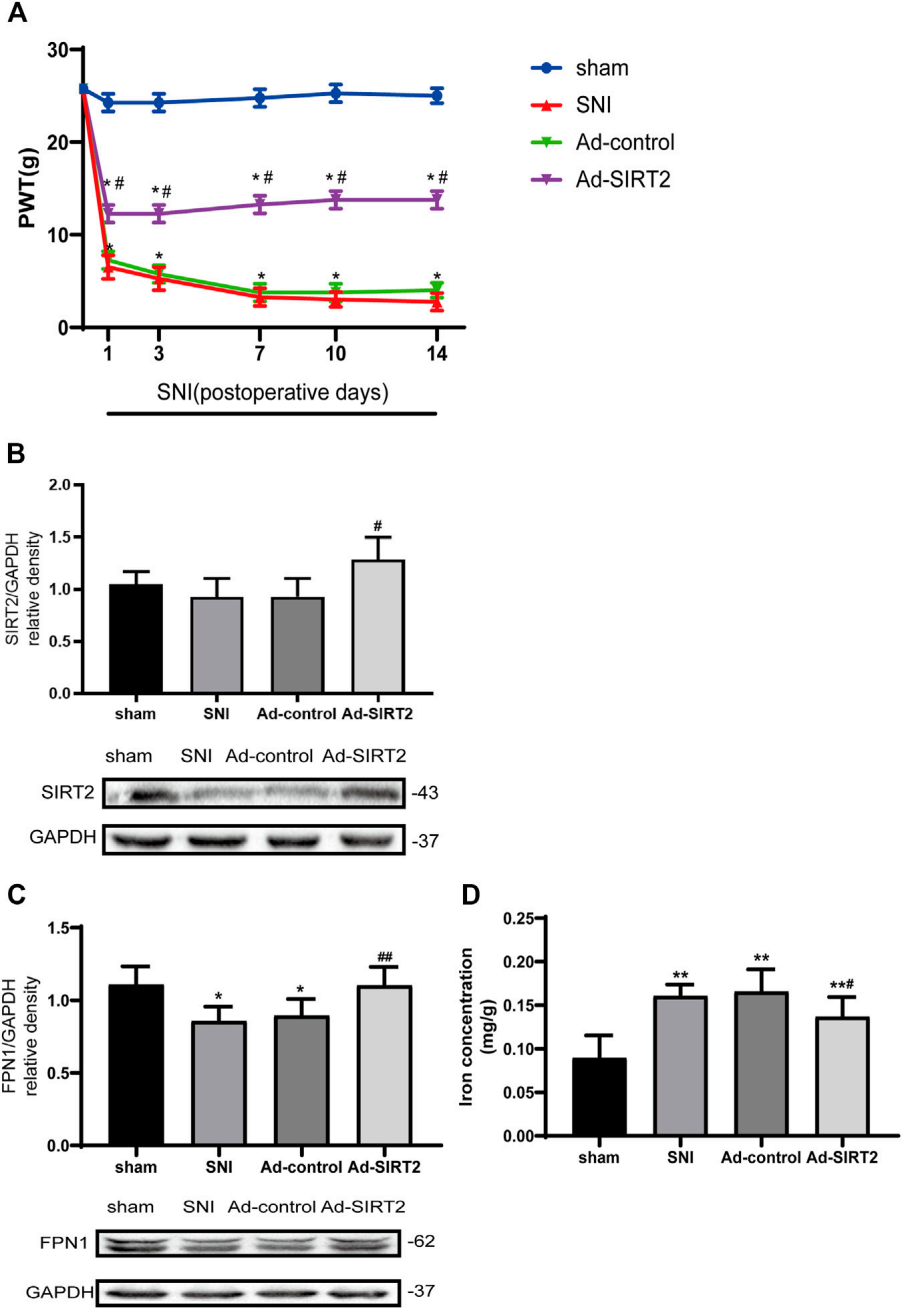
Intrathecal injection of SIRT2 adenovirus attenuated mechanical allodynia in SNI rats, enhanced the expression of FPN1, and reduced iron accumulation induced by SNI. **(A)** The effects of the intrathecal injection of Ad-SIRT2 or Ad-control on PWT in the sham group or SNI group. **(B)** Changes to SIRT2 expression in the spinal cord after an intrathecal injection of Ad-SIRT2 or Ad-control. **(C)** Changes to FPN1 expression in the spinal cord after an intrathecal injection of Ad-SIRT2 or Ad-control. The rats in the sham operation group were used as controls. **(D)** Changes to iron concentration in the spinal cord after an intrathecal injection of Ad-SIRT2 or Ad-control. Values are expressed as the mean ± SE (*n* = 5 per group). *p-values of <0.05 and **p-values of <0.01 for comparisons with sham rats or baseline values of the SNI rats (day 0); #*p*-values of <0.05 and ##*p*-values of <0.01 for comparisons with SNI or Ad-control rats. SNI, spared nerve injury; SIRT2, sirtuin 2; FPN1, ferroportin 1.

To better understand the mechanism of SIRT2 in NP regulation, the expression of SIRT2 in the spinal cord was upregulated by intrathecal injection of Ad-SIRT2, which significantly alleviated mechanical allodynia. The intrathecal injection of Ad-control did not alleviate mechanical allodynia in the SNI rats (Figure 4A). The Western blot analysis confirmed that compared with sham operation rats or SNI rats without intrathecal injection, the intrathecal injection of Ad-SIRT2 significantly enhanced the expression level of Ad-SIRT2 in the spinal cord of SNI rats (Figure 4B). These findings suggest that the overexpression of SIRT2 may relieve NP induced by SNI.

Next, we investigated the impact of SIRT2 on FPN1, a transmembrane protein that controls the efflux of iron. The overexpression of SIRT2 markedly increased the expression of FPN1 protein in the spinal cord of the SNI rats (Figure 4C). These results suggest that SIRT2 may alleviate NP by upregulating the expression of FPN1.

Moreover, compared to those in the sham or Ad-control rats, iron concentration in the spinal cord was significantly elevated in the SNI rats. In contrast, the iron content in the Ad-SIRT2 group was markedly reduced (Figure 4D). These findings suggest that FPN1 may participate in the regulation of intracellular iron content. The overexpression of SIRT2 could reverse the reduction of FPN1 caused by SNI and then allow the efflux of iron accumulated in the cell induced by SNI, thereby reducing the intracellular iron content and alleviating NP.

### SIRT2 Adenovirus Decreased Lipid Peroxidation Induced by SNI in the Spinal Cord

**FIGURE 5:**
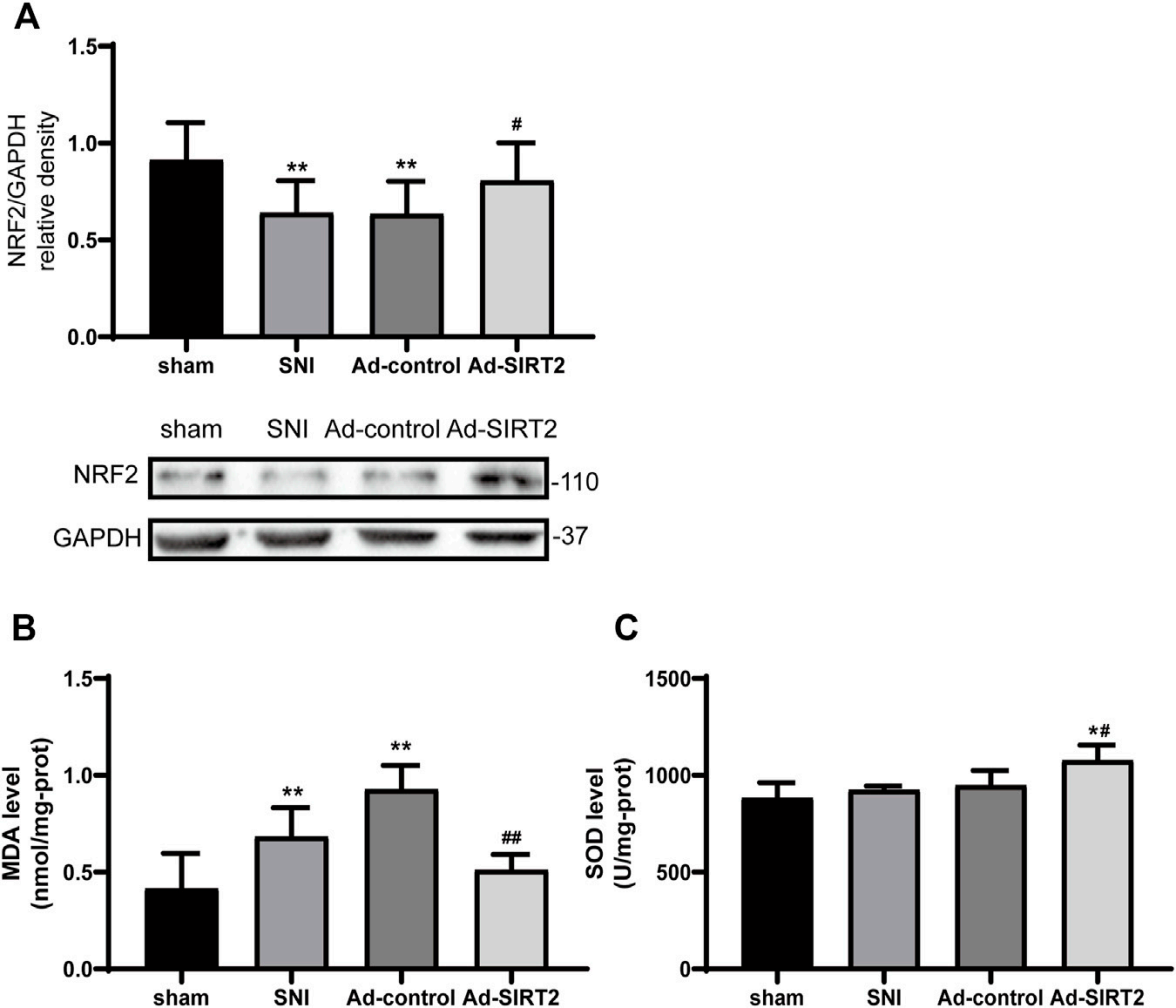
Intrathecal injection of SIRT2 decreases lipid peroxidation in the spinal cord of SNI rats. **(A)** Changes to NRF2 expression in the spinal cord after an intrathecal injection of Ad-SIRT2 or Ad-control. The rats in the sham operation group were used as controls. Changes to MDA **(B)** and SOD **(C)** expression in the spinal cord after an intrathecal injection of Ad-SIRT2 or Ad-control. The rats in the sham operation were used as the control group. Values are expressed as the mean ± SE (*n* = 5 per group). *p-values of <0.05 and **p-values of <0.01 for comparisons with sham rats or baseline values of the SNI rats (day 0); #*p*-values of <0.01 and ##*p*-values of <0.01 for comparisons with SNI or Ad-control rats. SNI, spared nerve injury; SIRT2, sirtuin 2; NRF2, nuclear factor erythroid 2-related factor 2; MDA, malondialdehyde; SOD, antioxidant enzyme superoxide dismutase.

The expression of NRF2 and concentration of MDA in the SNI group were lower and higher than those in the sham group, respectively (Figures 5A,B). Meanwhile, the concentration of SOD matched that of NRF2 in the SNI rats (Figure 5C). The overexpression of SIRT2 increased the expression of NRF2 and SOD (Figures 5A,C) and reduced the concentration of MDA in the spinal cord in the Ad-SIRT2 group (Figure 5B). These findings suggest that lipid peroxidation induced by SNI can be inhibited by SIRT2 overexpression.

### SIRT2 Adenovirus Upregulated the Level of GPX4 and Decreased the Level of ACSL4

**FIGURE 6:**
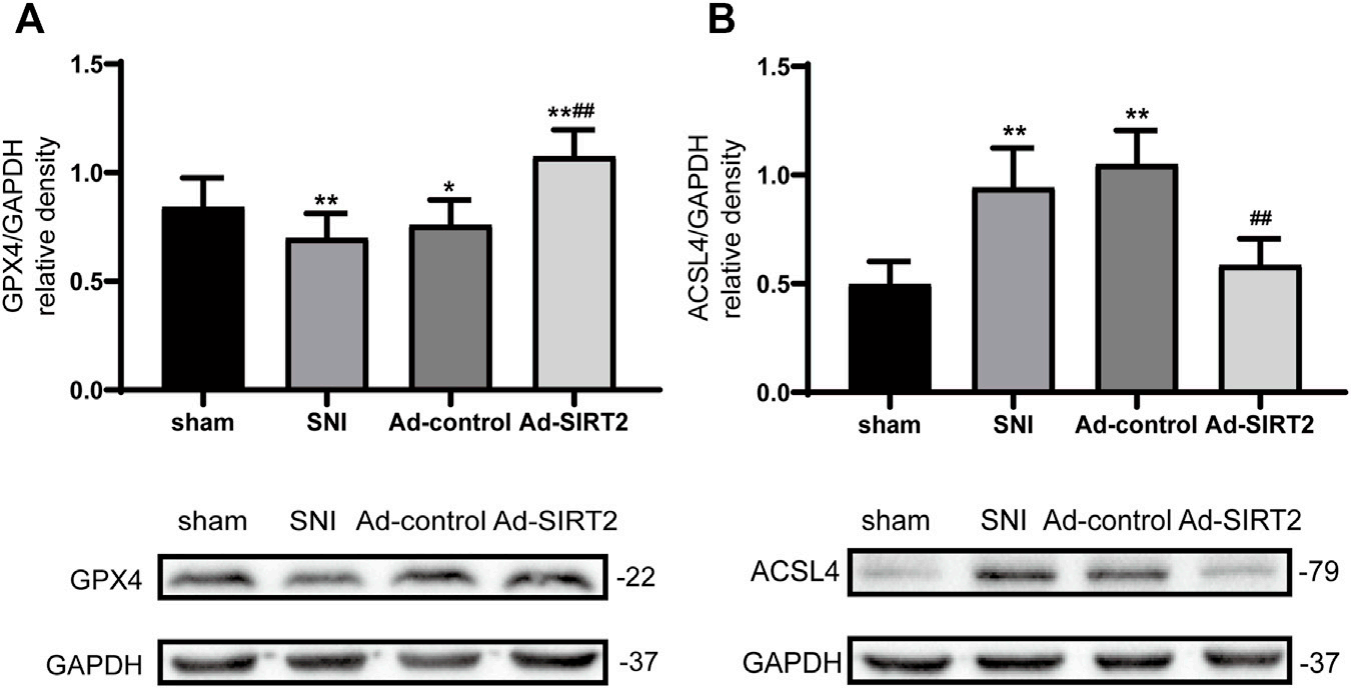
Intrathecal injection of SIRT2 increased the levels of GPX4 and decreased the levels of ACSL4. **(A)** Changes to GPX4 expression in the spinal cord after an intrathecal injection of Ad-SIRT2 or Ad-control. **(B)** Changes to ACSL4 expression in the spinal cord after an intrathecal injection of Ad-SIRT2 or Ad-control. The rats in the sham operation group were used as controls. Values are expressed as mean ± SE (*n* = 5 for each group). *p-values of <0.05 and **p-values of <0.01 for comparisons with sham rats or baseline values of the SNI rats (day 0); ##*p*-values of <0.01 for comparisons with SNI or Ad-control rats. SNI, spared nerve injury; SIRT2, sirtuin 2; GPX4, glutathione peroxidase 4; ACSL4, anti-acyl-coenzyme A synthetase long-chain family member 4.

Western blotting results showed that the expression levels of GPX4 decreased in the SNI group (Figure 6A), and those of ACSL4 in the spinal cord increased (Figure 6B). Meanwhile, the intrathecal injection of Ad-SIRT2 upregulated GPX4 expression (Figure 6A) and downregulated ACSL4 expression in the SNI rats (Figure 6B). There was no significant change in the expression of GPX4 and ACSL4 in the SNI group and Ad-control group.

## Discussion

The main findings from this study are presented as follows: *1*) The expression of SIRT2 and FPN1 decreased in SNI rats. *2*) Both SIRT2 and FPN1 expression decreased in the spinal dorsal horn microglia of SNI rats. In addition, the expression of FPN1 decreased in the spinal dorsal horn neurons of SNI rats. *3*) An intrathecal injection of Ad-SIRT2 upregulated the expression of SIRT2 and FPN1 in the spinal cord and reduced iron accumulation, alleviating mechanical allodynia in SNI rats. *4*) The overexpression of SIRT2 inhibited lipid peroxidation and reversed the changes to ACSL4 and GPX4 levels in SNI rats, thereby suppressing ferroptosis.

The number of studies on ferroptosis has increased in recent years. Although the relationship between ferroptosis and NP has been described, some molecules affecting ferroptosis remain subject to research. SIRT2 may regulate oxidative stress (Singh et al., 2018); it is the only sirtuin protein that is mainly found in the cytoplasm although it may also be present in the mitochondria and nucleus (Vaquero et al., 2006). Recently, accumulating evidence has revealed a relationship between SIRT2 and nervous system diseases (Yuan et al., 2016b; Zhang and Chi, 2018; Zhao et al., 2021). SIRT2 can exert a neuroprotective effect on TBI by inhibiting ferroptosis (Gao et al., 2021). This study is the first to propose that SIRT2 may relieve NP by inhibiting ferroptosis in SNI rats.

Iron is very important for life function; it participates in many human body biosynthesis processes, such as the synthesis of hemoglobin and myoglobin; it affects respiration and energy metabolism and is closely related to immunity (Steinbicker and Muckenthaler, 2013). However, iron overload may directly produce excess ROS, causing cell death and inducing a series of harmful reactions, such as oxidative stress (Steinbicker and Muckenthaler, 2013; Sheng et al., 2020). Therefore, rehabilitating iron homeostasis may help treat NP. FPN1, also called iron regulatory transporter 1 or metal transporter protein 1, is a transmembrane iron export protein. It is widely distributed in various tissues of the body; it is also present on the surface of macrophages, hepatocytes, intestinal enterocytes, and placental cells (Yang et al., 2002; Donovan et al., 2005). FPN1 mRNA is expressed in the small intestine, placenta, spleen, liver, kidney, heart, muscle, lung, and brain (Abboud and Haile, 2000). Thus, the expression of FPN1 is likely related to intracellular iron accumulation. In the present study, the expression of FPN1 decreased significantly on days 7 and 10 following SNI, and intracellular iron content increased in SNI rats, whereas an intrathecal injection of Ad-SIRT2 reversed these changes and alleviated mechanical allodynia induced by SNI.

NRF2 plays a key regulatory role in antioxidant stress (Sun et al., 2016). Most antioxidant stress processes in the human body require transcriptional regulation of NRF2 (Kuang et al., 2020). The activation of NRF2 may counteract the oxidative stress caused by ROS. In addition, several studies have shown that the downregulation or inactivation of NRF2 promotes ferroptosis (Sun et al., 2016; Song and Long, 2020). Therefore, NRF2 may be considered an important indicator of oxidative stress.

The process of cell death caused by iron overload and lipid peroxidation is called ferroptosis (Tang et al., 2021). Lipid peroxidation is mainly induced by polyunsaturated fatty acids (PUFA). During ferroptosis, lipid peroxidation further produces some substances, including the MDA and 4-hydroxynonenal (Tang et al., 2021). Studies have confirmed that erastin (Dolma et al., 2003) and RSL3 (Yang and Stockwell, 2008) are the main inducers of ferroptosis. Erastin indirectly inhibits GPX4 by suppressing system xc^−^ and consuming GSH, while RSL3 directly inhibits GPX4 to promote apoptosis (Yang and Stockwell, 2016). Thus, GPX4 plays an important role in the mechanism of ferroptosis. Ding and his group have shown that the ubiquitination of GPX4 caused its degradation and activated ferroptosis (Ding et al., 2021). ACSL4 is essential in the synthesis and metabolism of PFUAs because the upregulation of ACSL4 increases the concentration of PUFAs, which creates conditions for the occurrence of ferroptosis (Doll et al., 2017; Tang et al., 2021). The inhibition of ACSL4 prevented brain ischemia in mice, while the upregulation of ACSL4 deteriorated ischemic brain injury resulting from ferroptosis (Cui et al., 2021). ACSL4 promotes neuronal death by promoting lipid peroxidation, thereby triggering ferroptosis (Cui et al., 2021). Therefore, GPX4 and ACSL4 are important regulators of ferroptosis. In fact, the level of GPX4 reduced and the level of ACSL4 increased dramatically in NP rats induced by CCI (Wang et al., 2021).

This study has some limitations. The PWT in the SNI group, compared with the sham group, was significantly decreased 1 day after SNI, and this decrease persisted for the entire observation period. However, significant reductions in protein levels of SIRT2 in the spinal cord were observed 10 days after SNI. This mismatch in timecourse suggests that downregulated SIRT2 in the spinal cord mainly accounts for later stage NP (chronic NP); other mechanisms might contribute to early stage NP (acute NP). Proteins which function downstream of SIRT2, such as NF-κB p65, NF-κB p53 and NRF2, and upstream regulatory proteins targeting SIRT2, such as AMPK, NADH, AK-1 (SIRT2 inhibitor), can be the scope for future studies. SIRT is a highly conserved deacetylase. Whether its regulation of NP is upregulated or blocked by acetylation remains unclear. The present study has demonstrated that SIRT2 can alleviate neuroinflammation in SNI rats; significant upregulation of GPX4 expression and significant downregulation of ACSL4 expression were observed in SNI rats, which were reversed by the intrathecal injection of Ad-SIRT2. The present study has revealed the potential of SIRT2 as a new therapeutic target for alleviating NP.

In conclusion, nerve damage causes the downregulation of SIRT2 expression in the spinal cord and decreases the expression of FPN1, which causes iron ions to accumulate in the cell, as they cannot be transported to the outside of the cell. This mechanism causes oxidative stress induced by iron overload, which stimulates ferroptosis. The overexpression of SIRT2 alleviates NP induced by SNI by upregulating the expression level of FPN1 in the spinal cord of rats, reducing lipid peroxidation caused by iron accumulation, and reserving the changes of GPX4 and ACSL4 levels to suppress ferroptosis in the spinal cord. This evidence suggests that SIRT2-targeted therapeutics may help relieve the symptoms of chronic NP in clinical practice.

## Data Availability

The original contributions presented in the study are included in the article/Supplementary Material, further inquiries can be directed to the corresponding author.
